# The promise of virtual and augmented reality for optimizing community engagement in driving health equity solutions

**DOI:** 10.1186/s12889-026-28409-7

**Published:** 2026-07-09

**Authors:** Zakaria Nadeem Doueiri, Ann Banchoff, Lina María Gómez-García, Carlene Foldenauer, Paula Guevara Aladino, Astrid N. Zamora, Olga L. Sarmiento, Abby C. King

**Affiliations:** 1 Present Address: Department of Epidemiology and Population Health, Community Engagement and Implementation Coordinator, Our Voice: Citizen Science for Health Equity Initiative, Stanford University School of Medicine, 1701 Page Mill Road, Palo Alto, CA 94304 USA; 2 School of Medicine, Universidad de los Andes, Bogotá, Colombia; 3 Cabrillo Unified School District, Half Moon Bay, CA USA; 4 San Mateo County Office of Education, Safe Routes to School, San Mateo, CA USA; 5 Department of Medicine (Stanford Prevention Research Center), Stanford University School of Medicine, Palo Alto, CA USA

**Keywords:** Citizen science, Community engagement, Mixed methods, Our Voice, Social determinants of health, Virtual reality, Augmented reality, Vulnerable populations, Health equity

## Abstract

**Background:**

A growing number of studies have highlighted the importance of data visualization when utilizing mixed-methods approaches within community-based participatory research (CBPR). One such CBPR-derived method – *Our Voice* – employs a citizen-science (CS) framework that leverages mobile technology to improve health outcomes for residents from diverse communities. Virtual reality (VR) and augmented reality (AR) are two cutting-edge technologies that have been cited as potential tools to enhance data visualization and solution-building processes in *Our Voice* and similar community-facing methods. However, their utilization in such projects remains limited. In the present study, we report the first integration of VR and AR into two *Our Voice* projects and examine their feasibility and perceived utility within community-engaged settings.

**Methods:**

VR/AR technology was integrated into two *Our Voice* projects: (1) a project with adult unpaid care workers in Ciudad Bolívar, Bogotá, Colombia, and (2) a project with high school students in Half Moon Bay, CA, United States. In both settings, 360-degree simulations were integrated into a newly refined five-step *Our Voice* method, which included an “Imagine” step. Post-surveys assessed community members’ attitudes toward VR/AR using a six-point Likert scale. Data were descriptively analyzed.

**Results:**

Research using the *Our Voice* method revealed the integration of VR/AR resulted in citizen scientists and local stakeholders reporting VR/AR enhanced their ability to visualize community-level facilitators and barriers to leading healthy lives. Additionally, participants reported an increase in confidence in the potential of VR/AR to facilitate the development of innovative community-facing solutions to various issues around their neighborhoods.

**Conclusions:**

The findings from these pilot investigations suggest that VR/AR holds the promise in contributing to relevant advocacy changes across diverse communities, stakeholders, and problem areas. Future studies should explore developing user-friendly software that bridge data visualization and digital solution-building for broader community and organizational engagement.

**Supplementary Information:**

The online version contains supplementary material available at https://doi.org/10.1186/s12889-026-28409-7.

## Introduction

Citizen science (CS) offers a promising mechanism to address persistent health inequities by amplifying the voices of diverse individuals and subsequently driving change in their communities [1, 2]. The *Our Voice* (OV) method is one such CS approach that utilizes technology to elicit collective community experiences and perspectives on environmental living conditions. The current four-step OV method - *Discover*,* Discuss*,* Activate*, and *Change* - has demonstrated its success as an effective evidence-based approach to leverage data to drive positive change across numerous projects across the globe. In past OV projects, some changes have been short-term, such as campaigns aimed at organizing trash pick-ups, while others have led to longer-term ripple effects, such as pedestrian-friendly changes in local built environment infrastructure [3–6].

The OV research framework emphasizes community voices and experiences, whereby research personnel and local organizations work closely with community residents to identify *their* needs and tailor projects to address them. As part of this co-learning process, citizen scientists learn how to partner with community organizations and researchers to identify local issues and realistic solutions. This partnership also focuses on communicating findings in authentic and persuasive manners to key stakeholders [1]. One way of doing this is through the development of novel data visualization and solution-generation tools, such as innovative storytelling platforms, to enhance community engagement in research and present citizen science data in more informative and compelling ways [7]. A potential solution is to move past more typical two-dimensional forms of communication and harness the potential of virtual reality (VR) and augmented reality (AR), creating immersive visual and auditory experiences for citizen scientists and decision-makers which can allow them to more readily imagine issues and potential solutions in diverse settings (Appendix 1).

Although the intersection of VR/AR and citizen science is still in its early stages, previous studies have demonstrated the potentially transformative potential of these tools. For instance, students from Stanford University’s Virtual Human Interaction Lab (VHIL) utilized VR to showcase the impacts of human interaction on ocean acidification, bringing increased awareness among the Palauan National Congress [8]. Similarly, Columbia University’s Professor Courtney Cogburn developed an immersive VR experience called *1000 Cut Journey*, simulating scenarios contributing to the African American struggle in the U.S. Upon engaging students in the VR simulation, students became more “empathetic” and “aware” of various social justice issues [9]. Incorporating VR/AR into community-facing citizen science activities could lead to quicker consensus, swifter decision-making, and the ability to better visualize alternative interventions [7].

**Fig. 1:**
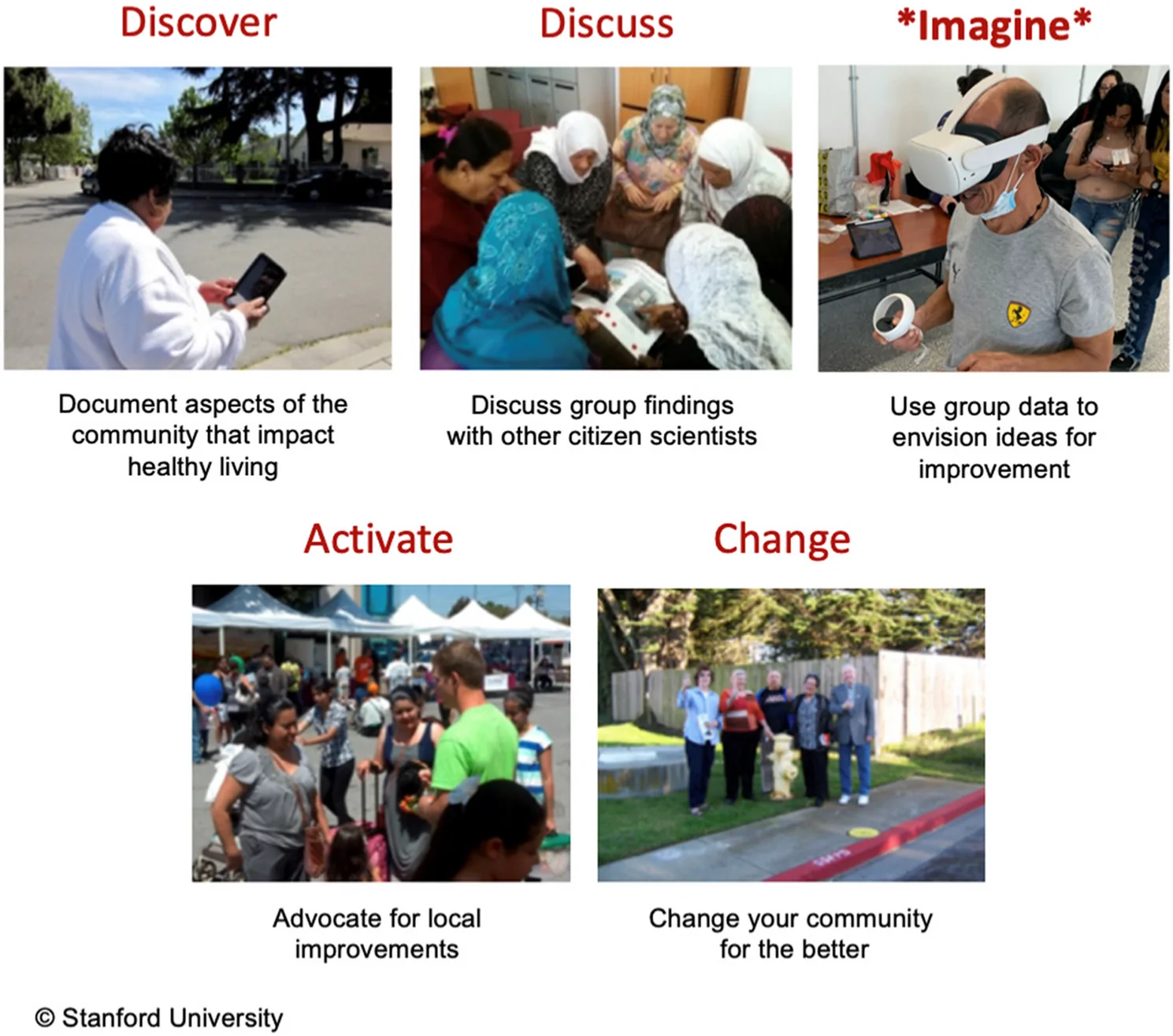
Refined *Our Voice* 5-Step Process

The two first-generation investigations that follow examine the impacts of VR/AR on citizen scientist engagement in a newly refined five-step *Our Voice* method which adds an “Imagine” step to the *Our Voice* process (Fig. 1). By integrating VR/AR into the evidence based *Our Voice* citizen science method, we aimed to (1) Examine their potential to enhance community engagement and better inform decision-making processes and (2) Explore the efficacy of VR/AR tools in facilitating actionable solutions to community health challenges in two distinct community settings. The first setting involved unpaid caregivers in Bogotá, Colombia, and the second engaged high school students at Half Moon Bay High School in Half Moon Bay, California, USA. These distinct settings were intentionally selected for this study, as the community collaborating organizations (Universidad de Los Andes and San Mateo County Office of Education, respectively) are long-standing partners of the *Our Voice* initiative, and expressed particular interest in piloting the additional “Imagine” step. Additionally, the community facilitators at each respective site had already built relationships and trust with the participants through previous community-engaged projects over the past few years, so they deemed it appropriate to choose these settings for this study. Since our two investigations were exploratory, our primary research questions were: (1) What is the feasibility of integrating VR/AR technology into the *Our Voice* method across diverse communities? and (2) How do community members and stakeholders perceive the usefulness of VR/AR to enhance data visualization, engagement, and solution-building? Our pilot investigations were designed as acceptability studies to inform future more rigorous adaptations.

## Methods

### The our voice citizen science method

The *Our Voice* method empowers community members to identify and engage with aspects of their local neighborhoods that either foster or impede a healthy lifestyle. By leveraging a user-friendly mobile application known as the Healthy Neighborhood Discovery Tool, developed by the Health Enabling Action Research & Technology Solutions (HEARTS) research laboratory at Stanford University [10], residents learn how to become “citizen scientists”. Using the Discovery Tool app, they collect geo-tagged photographs, audio/text narratives, and simple ratings that together capture their day-to-day experiences. The citizen scientists then collectively discuss their gathered data, distinguish and prioritize areas for modification, and communicate their data-driven suggestions to local stakeholders and decision-makers. This process, which historically consists of four distinct stages [10], paves the way for actionable and potentially sustainable improvements at the neighborhood level [11, 12]. See Fig. 1 below for a description of each step.

**Fig. 2:**
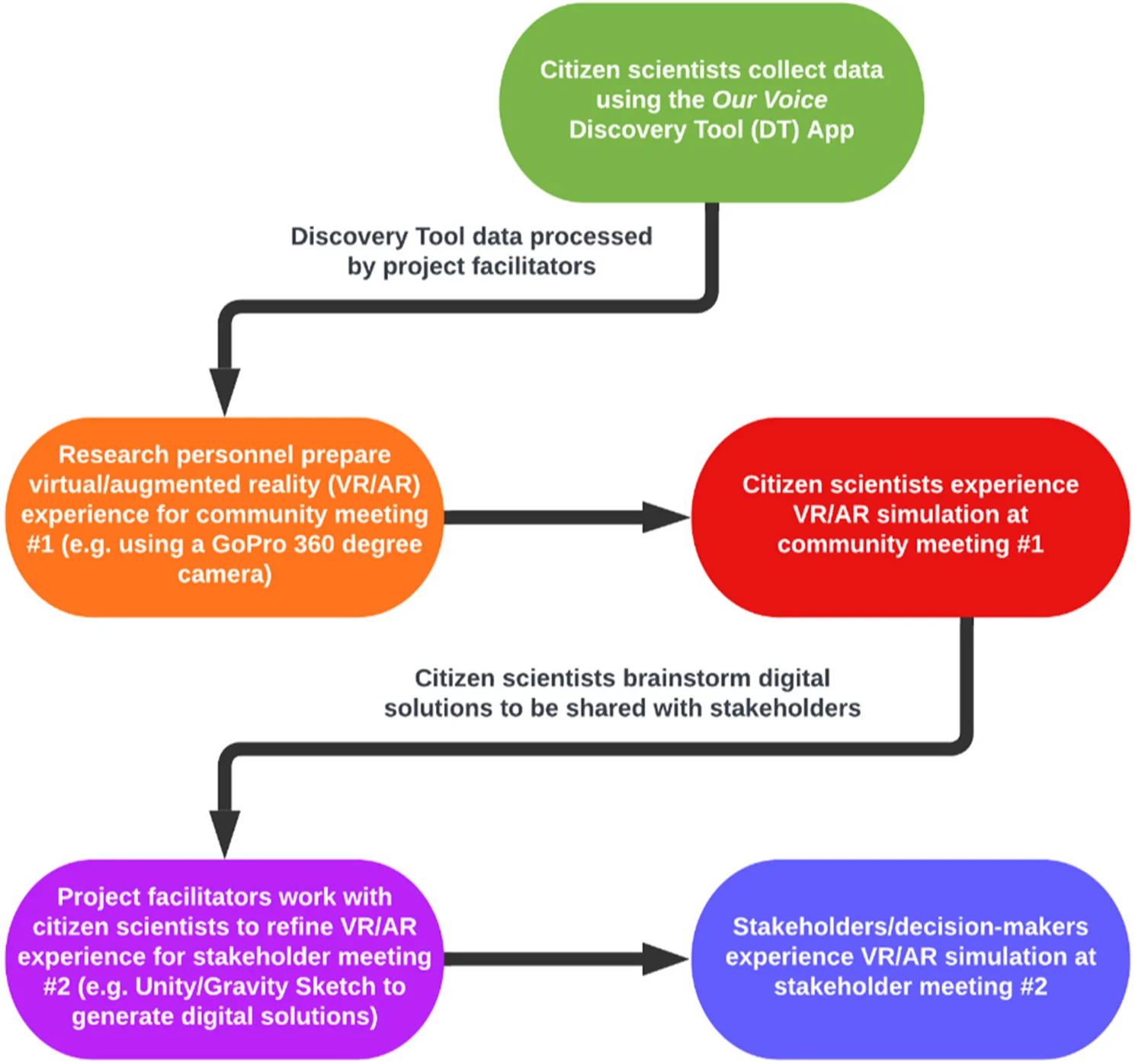
Virtual/augmented reality workflow in the *Our Voice* Method

- *Step 1. Discover*: The *Our Voice* method begins with the use of the previously described multilingual Discovery Tool mobile app, which allows citizen scientists to document local factors that positively or negatively impact their health/well-being. While conducting “walk audits,” citizen scientists use the Discovery Tool to capture geo-located photographs, rate pictures as positive, negative, or both, and describe their visual data through audio recordings or text comments.
- *Step 2. Discuss*: Following data collection, investigators consolidate data and generate PDF reports by transcribing audio recordings, rotating photographs, and blurring identifiable human features. Note that research personnel do not analyze the data. These data reports, which contain the citizen scientist-collected images, comments, geo-tagged map locations, and positive and/or negative ratings, are shared back with participants during facilitated community meetings. It is at these facilitated meetings that citizen scientists analyze their data, discuss their collective findings, and generate key themes and solutions. Participants reflect on each other’s perspectives, prioritize areas for improvement, and build consensus around solution feasibility.
- *Step 3. Imagine* (new step to Our Voice method): Historically, citizen scientists proceed to the next two steps of the *OV* method (*Activate* and *Change*), where community residents present their data and insights to key local decision-makers and stakeholders to enact change [11, 13]. However, in this study, VR and AR were added as an additional *Imagine* step which allowed participants and stakeholders to be immersed in the data through virtually “visiting” key hotspots and learning about the lived experiences of community members (Fig. 2). While the *Imagine* step can potentially take place before/after other steps of the *OV* method, in the two current investigations, it was inserted between the *Discuss* and *Activate* steps so users could experience the simulations prior to generating solutions.

In the current investigations, AR was used to expand the data visualization process, while VR was used to foster solution generation. After project facilitators processed the data collected in the *Discover* step, investigators returned to key locations and used a GoPro Max™ 360-degree camera to record footage at key hotspots identified by participants. Later, this footage was edited to include citizen scientists’ audio comments and formatted to be viewed on Oculus Quest 2™ Virtual Reality headsets. After the AR simulation was generated, citizen scientists experienced the interactive demonstration themselves at a meeting together and gave final confirmation that the simulation was reflective of their lived experiences prior to sharing the simulation with stakeholders. Citizen scientists also used Unity and GravitySketch 3D modeling VR software to either communicate with policy makers or design digital solutions. After refining the VR/AR simulation for use with community stakeholders, a second session was organized, providing stakeholders the opportunity to “virtually” return to dynamic points of interest from the citizen scientists’ walks (Fig. 2). All phases of Investigation #1 were reviewed and approved by the ethics committee of the Universidad de Los Andes (Acta No. 806–2017; Acta No. 1565–2022; 1571–2022, 1588–2022). All phases of Investigation #2 were reviewed and approved by the Stanford University Institutional Review Board (Protocol #45330).

- *Step 4. Activate*: After reviewing the full data set, the citizen scientists categorize their photos and narratives by theme, generate some potential solutions, and receive instruction from the community facilitators and Our Voice team in ways of communicating their information and insights to local policymakers and other relevant stakeholders (e.g., policymakers, law enforcement, architects).
- *Step 5. Change*: As part of this facilitated process, community members are coached on how to maintain close contact with each other, key decision-makers, and participating community organizations to reach benchmarks and work towards change in their communities. Our Voice research staff maintain close relationships with partnering organizations to systematically track and disseminate both the short and long-term changes that have occurred within each community. Following the stakeholder meeting that takes place in Step 4 “Activate,” citizen scientists, project facilitators, and stakeholders are encouraged to follow through with action items and report back the changes (ripple effects) that have been made in their communities.

#### Investigation 1

Unpaid caregivers in Ciudad Bolívar, Bogotá, Colombia.

## Methods

### Study setting and data collection

In Bogotá, Colombia’s capital of 7.9 million inhabitants, 35% of women spend over five hours a day on unpaid caregiving work compared to 8.6% of men [14]. In an effort to mitigate gender-related inequities in unpaid care work, the “Care Blocks’’ (in Spanish, “Manzanas del Cuidado”) public program was established by Bogota´s District Secretariat for Women [15]. Care Blocks offer multiple services for caregivers (e.g., education programs, physical activity classes, and wellness support), while simultaneously offering recipients access to care services and recreational activities [16]. Since the program is primarily aimed at supporting low-income caregivers, the first Care Block was strategically established in 2020 in Ciudad Bolivar. This area is a southwestern settlement of Bogotá characterized by a high poverty rate, with 10.9% of its population living in multidimensional poverty [17].

To better understand the barriers and facilitators that ease or hinder access and utilization of the Ciudad Bolívar Care Block facility, we employed the *Our Voice* citizen science method with a group of 21 caregivers between September 2021 and May 2023. Participants, called citizen scientists in this project, used the Discovery Tool for data collection over two weeks, responding to the guiding question, “What makes it easy or hard to get to/from your home to the Care Block program?” Complete study methods and results have been reported previously elsewhere [18]. The primary focus of this study was to investigate whether VR/AR enhanced citizen scientists’ abilities to visualize their data and go through the OV method.

### Preparing VR/AR simulation for community meeting #1 with citizen scientists

Following data collection, investigators used geotagged information to map the locations of the photos that were captured during the participants’ Discovery Tool walk audits. A total of 18 addresses from the locations with the highest frequencies of captured photos were selected to be displayed on an Oculus Quest 2™ Virtual Reality headset at the first community meeting. The VR application Wander™ - powered by Google Maps Street View™ - was used to map these locations.

### Community meeting # 1 with citizen scientists (discuss step)

Prior to the first community meeting, citizen scientists were randomly assigned to three groups. All three groups received copies of the photos captured from their Discovery Tool walk audits to collectively identify the most significant facilitators and barriers to accessing the Care Block and brainstormed solutions for the three most salient barriers in their respective groups. One of the three groups also received the augmented reality (AR) intervention, which allowed them to visualize their peers’ data using VR goggles. The virtual simulation was projected onto two external monitors so that the rest of the AR intervention group could see what the user was viewing on the headset. While each participant experienced the simulation of being at a problematic “hotspot” captured by their peer, the individual who took the photo(s) simultaneously explained the significance of that location to the group. At the end of the meeting, all three groups were brought back together, and a project facilitator from each group summarized the findings with everyone. All participants completed post-surveys assessing familiarity as well as opinions on augmented and virtual reality (Appendix 2). The survey used a six-point Likert scale, ranging from strongly disagree (1) to strongly agree (6), to rank the degree to which they agreed with the following statements: (1) “I can use technology to transform my community”; (2) “I know what augmented/virtual reality is”; and (3) “Augmented/virtual reality can be used to improve my community.”

### Preparing VR/AR simulation for community meeting #2 (activate step)

**Fig. 3:**
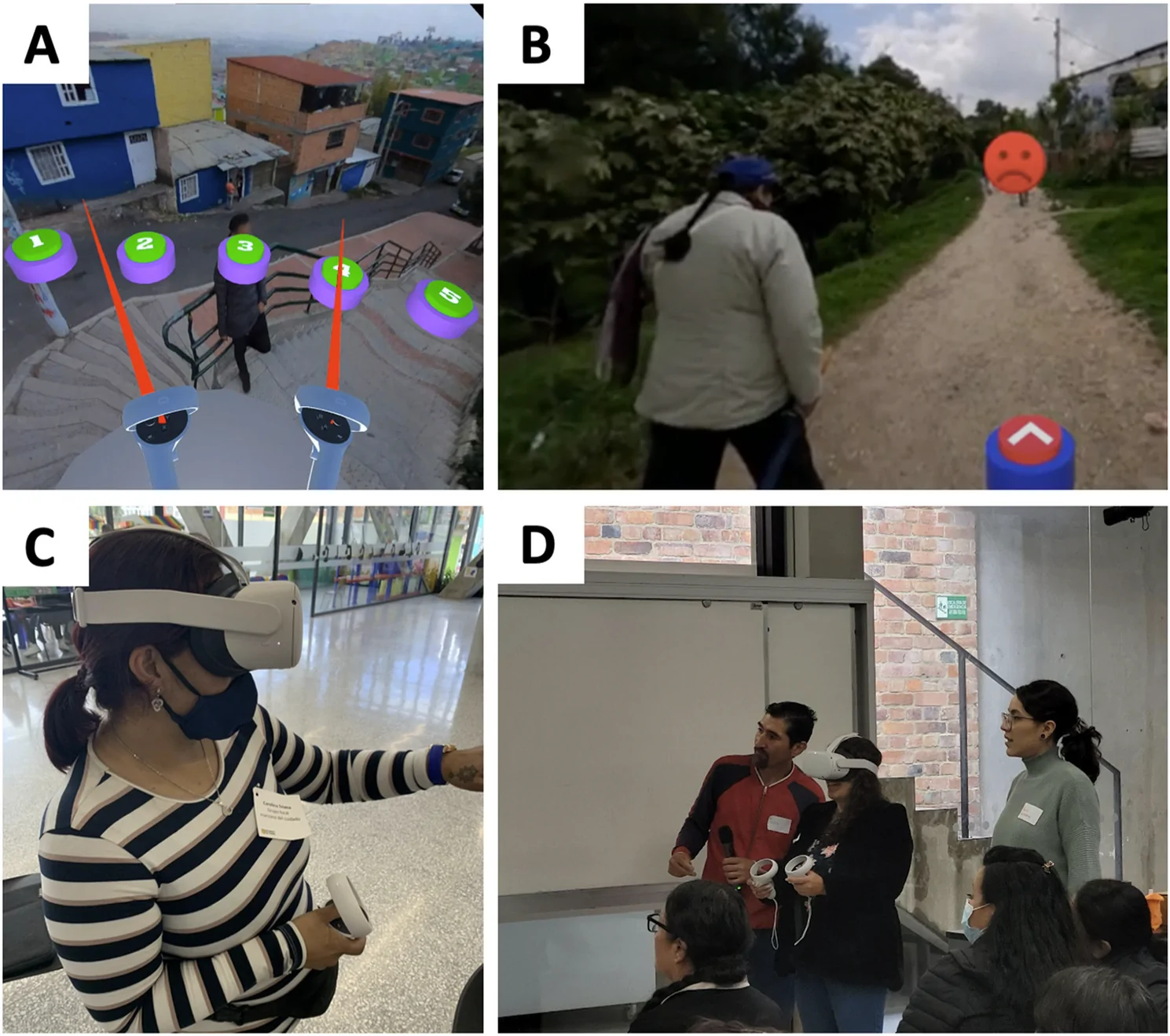
**A/B ** Virtual/augmented reality simulation in Bogotá, Colombia. **C ** Care worker trying virtual reality simulation. **D ** Care workers guiding decision-makers/stakeholders on VR/AR simulation

In preparation for the second community meeting with citizen scientists and stakeholders, Universidad de los Andes investigators created five automated VR/AR simulations (Fig. 3A and B). Three research personnel and two community members who participated with the data collection walk audits and community meeting returned to 15 locations around Ciudad Bolívar and used a GoPro Max™ 360-degree camera to capture photos and an iPhone to record sounds of the ambient environments. Locations were selected based on the themes with the highest frequency of photos taken by the citizen scientists (during the *Discover* step of the *Our Voice* method), and we used the Discovery Tool portal to reference the geotagged photos that citizen scientists captured to return to the location. After photographing these locations, an engineer at Universidad de los Andes designed an AR experience using automation algorithms to model and prototype the full simulation via Unity Gaming Services™ software (details are reported elsewhere [18]). The 360-degree images from these 15 locations were organized into five different augmented reality “paths” that users could select when using the Oculus Quest 2™ VR headset. Three photos and audio comments (chosen based on theme and audio quality) were used for each path, which were labeled by the citizen scientists via a green “happy” emoji face (facilitator) or red “sad” emoji face (barrier).

### Community meeting #2 (activate step)

For the stakeholder meeting, five citizen scientists were trained to use the Oculus Quest 2™ headset and to guide the others on the immersive simulation. Attendees experienced the augmented reality simulation after a brief introduction to the project (Fig. 3C and D). They later engaged in a dialogue (described elsewhere [18]), and all meeting attendees completed a post-intervention survey (Appendix 3). The survey used a different six-point Likert Scale that asked attendees to rank the degree to which they agreed with the following statements: (1) “Virtual reality is easy to use”; (2) “Virtual reality can be used to better visualize the problems of different communities”; (3) “Virtual reality enhances the creation of solutions for the community”; and (4) “Virtual reality can facilitate the dialogue between community and policymakers.”

### Data analysis

Given the small sample size and exploratory design, analyses were limited to descriptive statistics. The primary aim of this pilot study was to assess feasibility and acceptability of integrating VR/AR into the Our Voice method rather than to evaluate intervention effectiveness. Illustrative quotations from care workers and stakeholders are included to contextualize survey findings; however, formal qualitative coding procedures were not conducted given the exploratory design of this pilot study.

## Results

### Community meeting #1 with citizen scientists (discuss step)

A total of 21 citizen scientists (female = 20, male = 1) between the ages of 29–72 years (mean = 51.1 years) participated in the *Discover Step* data collection and the *Discuss Step* meeting. During the *Discover Step* walks, a total of 257 photos and audio recordings were collected and rated as facilitators (*n* = 79) or barriers (*n* = 178). During the *Discuss Step* meeting, citizen scientists collectively identified and prioritized the three most salient facilitators and barriers. Further details of these results are described elsewhere [18].

**Fig. 4:**
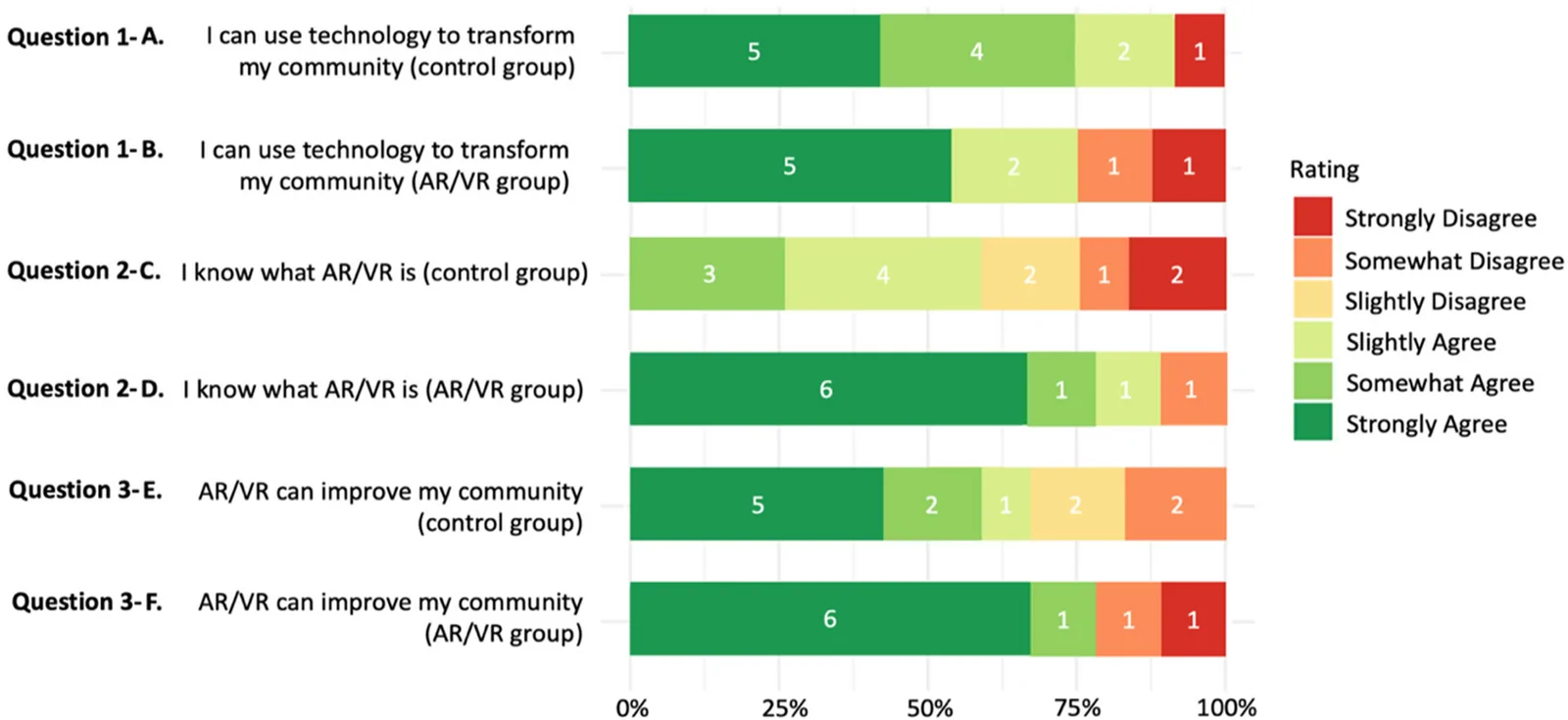
Post-survey responses from care workers in the experimental (*N* = 9) and control (*N* = 12) arms

In the *Discuss Step* community meeting, 9 of the citizen scientists were assigned to the virtual reality arm, and 12 were split evenly to each of the two regular (non-VR) arms (6 in each). When asked about the utility of technology to transform communities, 77.8% (*n* = 7) of the virtual reality arm agreed that technology could be used to positively improve their communities, while 91.7% (*n* = 11) of the control arm agreed with this statement (Fig. 4A and B). When asked whether participants knew what VR/AR was, 88.9% (*n* = 8) of participants in the experimental arm and 58.3% (*n* = 7) of participants in the control arm reported that they “Strongly, somewhat, or slightly agreed” with the prompt (Fig. 4C and D). Finally, when asked whether virtual reality could be used to improve their communities, 77.8% (*n* = 7) of the experimental arm agreed with the statement, while 66.7% (*n* = 8) of the control arm agreed (Fig. 4E and F).

### Community meeting #2 (activate step)

A total of 16 citizen scientists presented their findings to five professors from Universidad de Los Andes and eight policymakers from the Secretariats of Women, Habitat, Mobility, and Planning of Bogotá with the objective of improving access to the Care Block facility in Ciudad Bolívar. Additional details from the meeting are described elsewhere [18].

In the meeting, citizen scientists highlighted VR as a positive technology to immerse people in the data they collected, with one participant noting “It was a great experience being able to try virtual reality for the first time. Virtual reality takes you to the exact location and the audio helps you live the experience.” Policymakers also expressed their enthusiasm for using VR to inform decision-making processes by better understanding issues the community brings in shared spaces. A representative from the Urban Planning Department of Bogotá noted “Virtual reality allows you to understand the perspective of the people who live in a particular location. It [VR] brings urgency to improving many aspects of the built environment.”

As we have previously described [18], over two-thirds (67%) of citizen scientists thought VR was easy or moderately easy to use, while 76% believed that VR could enhance the OV method by allowing them to communicate more clearly the barriers of access to the Care Block. Moreover, most of the sample (81%) expressed that VR could help to create innovative solutions, and 71% considered that VR could facilitate the dialogue between citizen scientists and policymakers.

### Documented ripple effects (change step)

Following the *Activate Step* community meeting, members from Bogotá’s Secretariat of Planning invited the research team to present the VR experience at the Smart Cities Expo Bogotá 2023. Expo attendees expressed interest in the VR technology and after trying it, several individuals highlighted its potential to help citizens explore the city at the local level and understand the experiences of its inhabitants. Specifically, they noted that VR effectively illustrated the accessibility barriers caregivers face when commuting to the Care Block and also mentioned that VR fosters empathy by allowing users to relate these experiences to their own or those of their acquaintances.

#### Investigation 2

High school students in Half Moon Bay, California, USA.

## Methods

### Study setting and data collection

Half Moon Bay, California, USA, is located approximately 30 miles south of San Francisco on the coastline of the Pacific Ocean. In 2014, a Safe Routes to School (SRTS) program was implemented at numerous schools in the San Francisco Bay Area, including Half Moon Bay High School (HMBHS). The national SRTS program seeks to encourage active transportation and promote a culture of physical activity within the school community through safety education, infrastructural changes, and incentives [19].

**Fig. 5:**
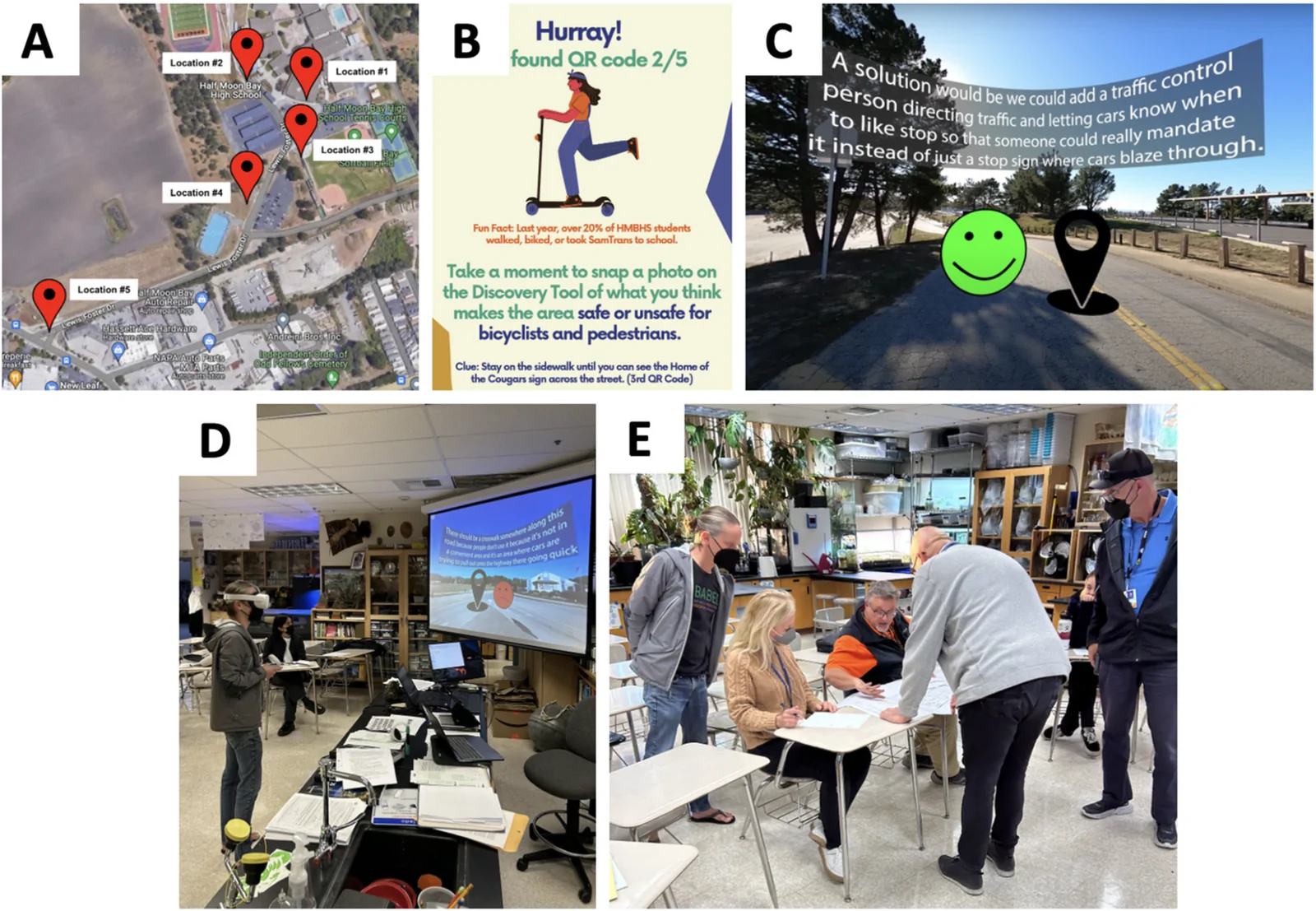
**A ** Map of locations at Half Moon Bay High School for data collection. **B ** Sample prompt after scanning QR code. **C ** Augmented reality experience at HMBHS. **D ** Teacher trying virtual reality simulation. **E ** Half Moon Bay High School/Cabrillo Unified school officials reviewing citizen scientist-generated data and solutions

While the SRTS program at HMBHS was successfully implemented prior to this investigation, the school reached out to *Our Voice* with interest in further engaging students in the documentation process regarding pedestrian and bicycle safety around the school. At HMBHS, the research team partnered with the Advanced Placement (AP) Environmental Science teacher to foster participation from students with firsthand experience walking, bicycling, and/or driving on campus and near the school. The 16 AP Environmental Science students (12 female and 4 male) used the *Our Voice* method to survey safety and accessibility issues related to walking and bicycling around HMBHS, including interaction with vehicles. At the beginning of the project, five key locations with known safety concerns were determined by HMBHS school administrators (Fig. 5A). Citizen scientists participated in a scavenger hunt where five groups of 3–4 students captured photos and comments in those key locations addressing the following guiding question: “What makes this path safe or unsafe for bicyclists and pedestrians?” Students scanned QR codes located at each location which prompted them to take photo-narratives, along with hints to help them find the next location (Fig. 5B**)**.

### Community meeting #1 with citizen scientists (discuss step)

At HMBHS, the first community meeting, which involved the student citizen scientist participants only, was conducted using the traditional *Our Voice* method through the distribution of the photos and comments collected via the *Our Voice* Discovery Tool application. Virtual and augmented reality were not used.

### Preparing VR/AR simulation for community meeting #2 with stakeholders (activate step)

Following the first community meeting with the student citizen scientists, investigators returned to HMBHS with a GoPro Max™ 360-degree camera and recorded 60-second video clips of each of the five locations. After video footage and audio recordings were gathered, Adobe Premiere Pro™ video editing software was used to connect them to the clips with the student narratives captured from the Discovery Tool (Fig. 5C). Subtitles were also added to each clip, and depending on whether a photograph captured a positive or negative feature, a green ‘happy’ emoji face (facilitator) or a red ‘sad’ emoji face (barrier) also popped up on the screen. Then, the video simulation was projected onto Oculus Quest 2™ goggles.

### Community meeting #2 with stakeholders (activate step)

The stakeholder meeting began with a review of the Discovery Tool walk audit data and the main themes developed by the students. Then, participants tested out the five-minute augmented reality experience, each viewing one of the five locations from the scavenger hunt “hotspots” (Fig. 5D). As users experienced the simulation, the rest of the participants in the meeting viewed the video and listened to the student narratives on a projector in the classroom. Participants completed pre- and post-surveys which assessed participants’ familiarity and experience with VR/AR using a 6-point Likert scale that ranged from strongly disagree to strongly agree regarding the following statements: (1) “Technology can be used to positively transform HMBHS”; (2) “I know what virtual reality is”; (3) “Virtual reality is intuitive and easy-to-use”; (4) “I can use virtual reality to better visualize issues relating to pedestrian/bicyclist safety at HMBHS”; and (5) “Virtual reality can be used to design solutions to various issues relating to pedestrian/bicyclist safety at HMBHS” (Appendix 4).

### Data analysis

Given the small sample size and exploratory design, analyses were limited to descriptive statistics. The primary aim of this pilot study was to assess feasibility and acceptability of integrating VR/AR into the Our Voice method rather than to evaluate intervention effectiveness.

## Results

### Community meeting #1 with citizen scientists (discuss step)

16 citizen scientists (female = 12, male = 4) between the ages of 16–18 years (mean = 16.5 years) from HMBHS participated in the *Discover Step* data collection and the *Discuss Step* community meeting. During the *Discover Step*, a total of 65 photos and audio recordings were collected (identified facilitators = 18; identified barriers = 44).

During the *Discuss Step*, students completed a solution-brainstorming worksheet (Appendix 5), spending ~ 7 min per location to review the full set of barriers and brainstorm potential solutions. A construction manager from the school district that HMBHS is in, Cabrillo Unified School District (CUSD), joined the community meeting to provide insights into the feasibility of the solutions being generated. After discussing their ideas, citizen scientists generated 34 strategies and solutions to address the challenges they noted at each location (Appendix 6).

### Community meeting #2 with stakeholders (activate step)

Two months after the first community meeting, the students presented their findings to school stakeholders with the objective of making the walking and biking routes to and from HMBHS safer. Nine participants attended the meeting: five high school student citizen scientists, a teacher, a HMBHS administrator, a CUSD administrator, and the CUSD construction manager.

After the AR data visualization was presented by the students, they transitioned from the *augmented* reality experience to a *virtual* reality experience. Due to time constraints, a project investigator referenced the students’ solutions in designing and showing some digital representations of those solutions via the virtual reality software *GravitySketch*™. Then the students discussed with the school officials the feasibility of proposed solutions after viewing the solutions on the VR headset (Fig. 5E).

**Fig. 6:**
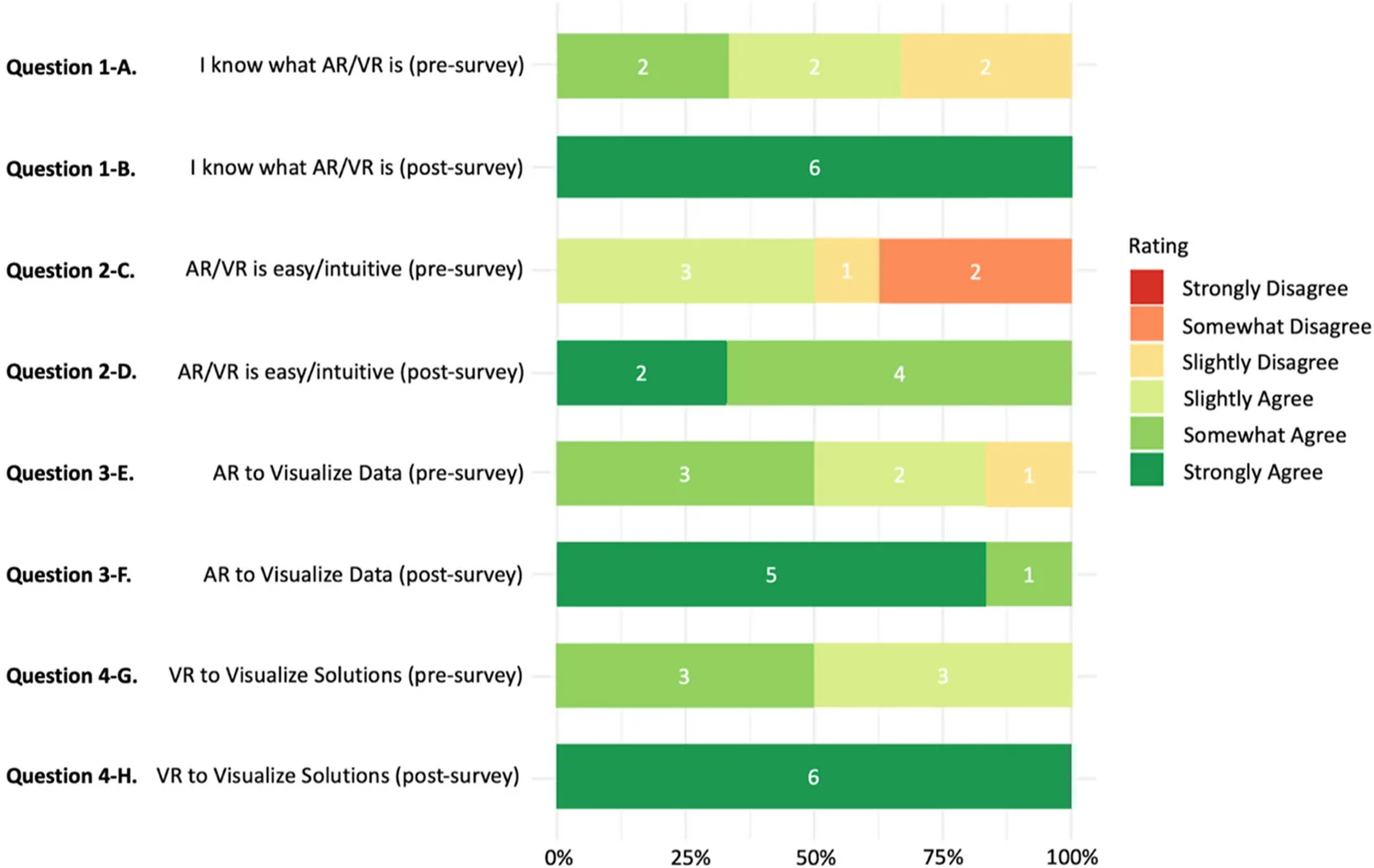
Pre- and post-survey responses from students at Half Moon Bay High School (HMBHS) (*N* = 6)

Pre- and post-surveys were administered to participants during the community stakeholder meeting before and after experiencing the VR/AR simulation. When asked whether participants knew what VR/AR was prior to experiencing VR/AR, 66.7% (*N* = 4) of participants responded, “Slightly or Somewhat Agree” and 33.3% (*N* = 2) of participants responded, “Slightly Disagree” (Fig. 6A). After experiencing the simulation, 100% (*N* = 6) responded “Strongly Agree” to this same question (Fig. 6B). Similar results were found for the question assessing the difficulty/accessibility of using VR/AR (Fig. 6C and D).

Additional survey items assessed whether participants believed AR could be used as a visualization tool (Fig. 6E and F) and whether VR could be leveraged to build solutions (Fig. 6G and H). Prior to experiencing VR/AR, when asked whether it could help visualize data, 83.3% (*N* = 5) of participants responded “Slightly or Somewhat Agree” and 16.7% (*N* = 1) responded “Slightly Disagree.” When asked whether VR could help design solutions, 100.0% (*N* = 6) of participants responded “Slightly or Somewhat Agree.” After experiencing the VR/AR intervention, all participants increased their confidence in using this technology to survey and intervene in their environment (83.3% (*N* = 5) of participants responded “Strongly Agree” for VR to support data visualization and 100.0% (*N* = 6) of participants responded “Strongly Agree” for VR to help in the design of solutions) (Fig. 6E-H).

## Discussion

At the core of the *Our Voice* method – and community-based participatory research more broadly – is the goal of getting residents excited about engaging in their *own* research in collaboration with scientists. Current *Our Voice* projects have successfully involved “non-scientists” in this process by bringing the voices of diverse community members to the forefront of the conversation [3, 5, 6, 20]. However, capturing the authentic, lived experiences of individuals can sometimes be a challenge. This study was the first of its kind to pilot the integration of a new *Imagine* step in the OV method to enrich data visualization for both community members and stakeholders using virtual and augmented reality. The findings of this study suggest that virtual and augmented reality may help bridge the gaps between gathering community perspectives, enhancing community engagement, and informing decision-making processes by helping to visualize those perspectives and design solutions in novel ways. In this manner, such technological advances can creatively encourage participants and policymakers to consider and compare other perspectives with their own and help facilitate dialogue between stakeholders to elicit change.

Participants in both investigations who used VR/AR increased in their understanding of the technology and experienced first-hand its relative ease of use. Moreover, they were able to realize the potential capabilities of these tools to visualize data and build digital solutions to address community-related determinants of health. Results from both investigations indicated that participants using VR/AR technology in conjunction with the Our Voice method reported feeling better equipped to improve their communities by the end of the study. However, it is important to note that the comparative effectiveness of the Our Voice method with and without VR/AR was not systematically tested, suggesting a need for further research to understand the impact of VR/AR on follow-through for action items. The next step in investigating the added strengths of VR/AR in this type of research-to-action citizen science method would be to experimentally compare the *Our Voice* method with and without the additional VR/AR methodology.

Participants in both investigations expressed enjoyment in having the autonomy to explore virtual and augmented reality simulations. During the first citizen scientist community meeting with caregivers in Investigation #1, participants enjoyed the opportunity to explore virtual and augmented reality simulations, however, two elderly participants had discomfort with the VR goggles, with one individual experiencing vertigo. We hypothesize this may explain the results in Fig. 4A/B, where a larger percentage of participants in the control arm reported being able to personally use technology to positively improve their communities compared to the VR/AR intervention arm. Despite this, the VR group enthusiastically requested more time to engage with the VR system, indicating their eagerness to contribute to the theme-identification and solution-building aspects of the meeting. At HMBHS, students had a similar reaction to the technology. These results are in line with dozens of previous studies that suggested meaningful effects of immersive virtual reality on individuals’ attitudes related to excitement and enthusiasm to learn new information about themselves and their surroundings [21, 22]. While the overarching goal of the Our Voice method is to activate residents to create positive change in their local communities, a key ingredient that has led to the success of this solution-oriented methodology in over 100 projects throughout the globe has been its particular emphasis on promoting self-efficacy among community members.

In both investigations, participants who experienced the VR/AR simulation reported increased confidence to not only visualize issues around their communities but also make meaningful changes as well. Given these initial responses, it is possible that the utilization of VR/AR may contribute to increased self-efficacy and engagement in community-engaged projects throughout the OV or similar participatory research processes. These findings support the literature that has previously found associations between individuals’ exposure to virtual reality and increased feelings of learning agency and self-efficacy [23].

Since a key element of citizen science is the democratization of research, and given that mistrust from community members toward researchers and other stakeholders persists, particularly in minority and low-SES populations [24], this technology may also aid in the recruitment of populations less likely to engage in community-based research by getting individuals excited about contributing to project goals through learning how to experience and use such novel technologies.

Given the diversity of occupation, gender, and age backgrounds of participants across the two investigations, the study results illustrate that VR/AR has the potential to make research more participatory on a broader scale. By involving community members in the data collection and evaluation processes in creative and innovative ways, VR/AR may help to break down the barriers that constrict *who* has access to research data, resources, and results.

### Limitations

While technology knowledge and positive perceptions about VR/AR increased in both investigations, it is important to note some of the limitations of this study. First, sample sizes in both investigations were too small to evaluate statistical significance. Similarly, the gender split between male and female citizen scientists in both investigations was heavily skewed toward female participants. In the first investigation, this reflects the real-world demographics of the Care Block facility, which primarily serves women caregivers—a pattern consistent with global caregiving trends in which women disproportionately assume caregiving roles. In the second investigation, the participating high school class had approximately three times as many female students as male students. Future studies may consider broader recruitment strategies to promote greater gender balance, particularly in settings where more equal representation is feasible and appropriate.

Additionally, utilization of AR did not occur at HMBHS until the community meeting with stakeholders due to the short turnaround between data collection and discussion. Given that this project took place in an AP Environmental Sciences class, investigators were limited on the dates they could work with students. Such constraints are expected in such community-facing participatory research methods, which emphasize the importance of being community-responsive, thus adapting the approach based on specific project preferences and constraints [2].

As noted in the study designs for Investigation #2 at HMBHS, most but not all participants at the second community meeting wanted to experience the virtual reality simulation. Consequently, for Investigation #2, we are unable to determine whether positive perceptions of VR/AR are related to the use of the technology specifically or rather from engaging in the traditional *Our Voice* community meeting participation more generally. As noted above, the next step in this line of research is to experimentally test the benefits of adding VR/AR technology to the traditional *Our Voice* citizen science process to determine what additional advantages might accrue from adding this method, as well as better determining its limitations.

With respect to the solution-building utilization of VR/AR, we initially sought participants to be more involved in manipulating their virtual simulations. However, the learning curve of being trained on how to manipulate the virtual environment, paired with time constraints, made it challenging for participants to design their own solutions. Future research may consider testing more user-friendly 3D modeling software. Another type of solution would be to employ a “train-the-trainer” approach whereby volunteers from the community settings involved are trained on relevant applications of VR/AR for their community, and then can in turn train others from their community.

### Future directions

Taken together, citizen scientists and community stakeholders can benefit greatly from using different VR/AR protocols (e.g., Appendix 7). By sharing these diverse approaches to integrating VR/AR into the *Our Voice* method and similar participatory research approaches, participants can learn from each other’s experiences and adapt a set of best practices to fit their specific contexts. This holds promise for not only enhancing the data visualization and solution-building process, but also contributing to a more collaborative and dynamic approach to addressing community-related determinants of health.

In addition to the potential impacts of virtual and augmented reality on data visualization and solution-building, the use of these technologies can contribute to the sharing of experiences across both research and community groups. Unique virtual reality protocols may offer varied perspectives and approaches to the same problem, leading to a richer understanding of community-related health determinants. For example, in Investigation #1 in Bogotá, Colombia, an engineer used *Unity*™ to design the VR simulation for the stakeholder meeting, which provided stakeholders the ability to experience different VR “paths” based on specific themes of interest. Meanwhile, the VR experience for the students at HMBHS was developed using Adobe Premiere Pro, generating a singular VR simulation where stakeholders were able to visualize the five locations with known safety concerns and suggest various solutions. The different VR experiences offered a range of community-engaged visualizations that resonated with different stakeholders based on their professional backgrounds.

Finally, when considering the feasibility of formally integrating VR/AR into this type of participatory citizen science data collection and analysis process, there are several characteristics of the technology to take into consideration. The first is determining how to capture diverse environments for display on the VR goggles platform without spending too much time or resources depicting a particular virtual environment. Related to this issue is the challenge at times of budgetary constraints. Given that cost may be a barrier to entry, VR/AR may not be a feasible technology to add to every project. The *Our Voice* Initiative regularly lends $30 USD-Android TracFones to collaborators who are implementing projects and need devices to go on walk audits. A similar system can potentially be used to lend VR goggles and 360-degree cameras to partners interested in incorporating the VR/AR technology into their own community projects. Alternatively, project facilitators may consider purchasing a VR headset that does not have a built-in processing system (e.g., fitting a mobile device into the shell and viewing the VR experience through the phone screen). These headsets can serve as cost-effective alternatives for communities that do not have the resources to purchase more expensive headsets.

## Conclusions

In summary, the two investigations undertaken in this study integrated VR and AR into a participatory citizen science method, with encouraging findings. Across both studies, citizen scientists and local stakeholders reported that VR/AR enhanced their ability to visualize community-level barriers and facilitators to leading healthy lives. The technology was also perceived to facilitate the development and brainstorming of solutions to address various social determinants of health around their neighborhoods. Based on these results, we recommend that further systematic investigation of the advantages of VR/AR technologies in aiding real-world visualizations of both community-based challenges and solutions occur.

## Supplementary Information

Supplementary Material 1.

## Data Availability

Data are provided within the manuscript.

## Abbreviations

AR: Augmented reality
CBPR: Community-based participatory research
CS: Citizen science
CUSD: Cabrillo Unified School District
HMBHS: Half Moon Bay High School
OV: Our Voice
SRTS: Safe Routes to School
VR: Virtual reality
