# Supplementary material for: The promise of virtual and augmented reality for optimizing community engagement in driving health equity solutions
Source: BMC Public Health. 2026 Jul 9;26:2868. doi: 10.1186/s12889-026-28409-7 (PMC13647544; doi:10.1186/s12889-026-28409-7)
Supplement: Supplementary file 1 [file 12889_2026_28409_MOESM1_ESM.docx]

**APPENDICES**

**Appendix 1:** *Our Voice* Research Framework with Additional VR/AR Component (Adapted from King AC. Theory’s role in shaping behavioral health research for population health. Inter J Behav Nutr Phys Activ, 2015, 12:146, DOI 10.1186/s12966-015-0307-0)

**Appendix 2:** Virtual Reality Post-Survey at Manzanas del Cuidado Community Meeting #1

1) I can use technology to transform my community.
   a) Strongly Disagree
   b) Somewhat Disagree
   c) Slightly Disagree
   d) Slightly Agree
   e) Somewhat Agree
   f) Strongly Agree
2) I know what virtual reality is.
   a) Strongly Disagree
   b) Somewhat Disagree
   c) Slightly Disagree
   d) Slightly Agree
   e) Somewhat Agree
   f) Strongly Agree
3) Virtual reality can be used to improve my community.
   a) Strongly Disagree
   b) Somewhat Disagree
   c) Slightly Disagree
   d) Slightly Agree
   e) Somewhat Agree
   f) Strongly Agree

**Appendix 3:** Virtual Reality Post-Survey at Manzanas del Cuidado Stakeholder Meeting #2

1) What is your name?

_______________________________________

2) What is your gender?
   a) Man
   b) Woman
   c) Non-binary
   d) Other ___________
3) In which locality and town do you live in? (e.g. La Candelaria, Las Aguas)

_______________________________________

4) Which sector are you a part of?
   a) Public sector
   b) Academic sector
   c) Community sector
   d) Other ___________

Mark each of the following statements:

5) There are clear action items and ways I can improve access to the Manzanas del Cuidado community center in Ciudad Bolívar.
   a) Strongly Disagree
   b) Somewhat Disagree
   c) Slightly Disagree
   d) Slightly Agree
   e) Somewhat Agree
   f) Strongly Agree
6) Virtual reality is easy to use.
   a) Strongly Disagree
   b) Somewhat Disagree
   c) Slightly Disagree
   d) Slightly Agree
   e) Somewhat Agree
   f) Strongly Agree
7) I can use virtual reality to better visualize the problems in different communities.
   a) Strongly Disagree
   b) Somewhat Disagree
   c) Slightly Disagree
   d) Slightly Agree
   e) Somewhat Agree
   f) Strongly Agree
8) The virtual reality experience has the potential to create solutions for the community.
   a) Strongly Disagree
   b) Somewhat Disagree
   c) Slightly Disagree
   d) Slightly Agree
   e) Somewhat Agree
   f) Strongly Agree
9) Virtual reality can help facilitate dialogue between the community and decision-makers.
   a) Strongly Disagree
   b) Somewhat Disagree
   c) Slightly Disagree
   d) Slightly Agree
   e) Somewhat Agree
   f) Strongly Agree
10) Use this space to share any additional comments about today’s meeting.

_______________________________________

**Appendix 4:** Virtual Reality Pre and Post-Survey at Half Moon Bay High School Stakeholder Meeting #2

1) Technology can be used to positively transform HMBHS.
   a) Strongly Disagree
   b) Somewhat Disagree
   c) Slightly Disagree
   d) Slightly Agree
   e) Somewhat Agree
   f) Strongly Agree
2) I know what virtual reality is.
   a) Strongly Disagree
   b) Somewhat Disagree
   c) Slightly Disagree
   d) Slightly Agree
   e) Somewhat Agree
   f) Strongly Agree
3) Virtual reality is intuitive and easy-to-use.
   a) Strongly Disagree
   b) Somewhat Disagree
   c) Slightly Disagree
   d) Slightly Agree
   e) Somewhat Agree
   f) Strongly Agree
4) I can use virtual reality to better visualize issues relating to pedestrian/bicyclist safety at HMBHS.
   a) Strongly Disagree
   b) Somewhat Disagree
   c) Slightly Disagree
   d) Slightly Agree
   e) Somewhat Agree
   f) Strongly Agree
5) Virtual reality can be used to design solutions to various issues relating to pedestrian/bicyclist safety at HMBHS.
   a) Strongly Disagree
   b) Somewhat Disagree
   c) Slightly Disagree
   d) Slightly Agree
   e) Somewhat Agree
   f) Strongly Agree

**Appendix 5:** Solution-Brainstorming Worksheet for Community Meeting #1 at HMBHS

**Appendix 6:** Student-Generated Solutions to Safety Concerns at HMBHS

9/29/22 – Guiding Question: How can we enable more students to walk and bike to school safely?

Recap of Student Ideas (5 Groups)

Location #1: Drop-off/Pick-up Zone in front of the HS

-	Expand the current drop-off/pick-up zone to two lanes (one lane for drop off and

second lane for driving by). (X4 groups)

-	Make the drop-off/pick-up curb longer.

-	Remove the planter box to make more room for the sidewalk.

-	Put crosswalks at the end of the parking lot (drop off zone).

Location #2: Upper Student Parking Lot Area

-	Put a crosswalk to Cougar Park and make a pathway around the upper parking lot.

-	Move bike parking to the back of the parking lot (northwest corner).

-	Paint a crosswalk from the upper parking lot to campus.

-	Put a crosswalk at the flagpole.

-	Have a bike route that leads to the bike rack.

-	Add a bike lane to the existing walkway (X2 groups)

-	Implement Crossing Guard Program (possibly students)

Location #3: Top of Lewis Foster Drive

-	Put in signs to alert drivers of the crosswalk (at the top of Lewis Foster Drive).

-	Repaint STOP lettering, limit lines, and Crosswalks.

-	Put in a Stop sign at the top of Lewis Foster Drive.

-	Have a crossing guard at the top of Lewis Foster Drive crosswalk.

-	Build a bike lane or walkway on the east side of Lewis Foster drive.

-	Repair/replace solar lights that are not working along Lewis Foster Drive.

-	Add lights at the stairway to make it safer.

-	Redo stairway to lower lot to make it safer.

-	Consider making the lower lot a drop-off area.

Location #4: Mid-way Lewis Foster Drive (upper lot driveway intersects with Lewis Foster Dr.)

-	Build a walkway and bike lane around the <upper> parking lot and connect it to

the sidewalk and walkway along Lewis Foster Drive (to avoid driver/pedestrian/bicycle conflict at the driveway to upper lot and Lewis Foster Dr.).

-	Put the bike lane on the opposite side. Prevent bicyclists from going up the lot.

<driveway to upper parking lot?>

-	Move the crosswalk to the top of the hill at the upper parking lot, so students can

cross, and then go down the hill to the Lewis Foster Drive Pathway <would need a walkway along the driveway from the upper lot down to Lewis Foster Drive>.

-	Widen the sidewalk along Lewis Foster Drive.

-	Move the sidewalk to the other side.

-	Use the lower parking lot as an alternative pick-up/drop-off area.

-	More roadside lights for better visibility (night events).

Location #5: Bottom of Lewis Foster Drive at North Main Street

-	The crosswalk at the bottom of the hill <at North Main Street> is long and

confusing. Move the crosswalk further up Lewis Foster Drive - mid-block. (X5 groups)

-	…and take out the crosswalk and yield sign at Lewis Foster Drive and Main

Street.

-	Consider speed bumps (or little yellow bumps) to slow traffic going up/down

Lewis Foster Drive.

-	Provide traffic control (crossing guards)

-	Pave the road that leads to 92 and put in a traffic light to provide a second

exit/entrance to the school. CITY

-	Put in a Traffic Light <at Lewis Foster Drive and North Main Street>. CITY

-	Put North Main Street Crosswalk on the other side of Lewis Foster Drive so

students don’t have to cross over Lewis Foster Drive. CITY

**Appendix 7:** Virtual Reality Usage Guide for Manzanas del Cuidado Project (developed by *Our Voice* collaborators at Universidad de Los Andes and Stanford University)
